# Remodelling and dysfunction of the sinus node in pulmonary arterial hypertension

**DOI:** 10.1098/rstb.2022.0178

**Published:** 2023-06-19

**Authors:** Sunil Jit R. J. Logantha, Tomoko T. Yamanushi, Mais Absi, Ian P. Temple, Hideaki Kabuto, Eiichiro Hirakawa, Gillian Quigley, X. Zhang, Alison M. Gurney, George Hart, Henggui Zhang, Halina Dobrzynski, Mark R. Boyett, Joseph Yanni

**Affiliations:** ^1^ Department of Cardiovascular and Metabolic Medicine and Liverpool Centre for Cardiovascular Science, University of Liverpool, Liverpool L7 8TX, UK; ^2^ Division of Cardiovascular Sciences, University of Manchester, Manchester M13 9PL, UK; ^3^ Department of Physics and Astronomy, University of Manchester, Manchester M13 9PL, UK; ^4^ Graduate School of Health Sciences, Kagawa Prefectural University of Health Sciences, Takamatsu City, Kagawa 761-0123, Japan; ^5^ Department of Anatomy, Jagiellonian University Medical College, Kraków 31-008, Poland; ^6^ Faculty of Life Sciences, University of Bradford, Bradford, West Yorkshire BD7 1DP, UK

**Keywords:** pulmonary arterial hypertension, heart failure, sinus node, ion channels, cardiac remodelling

## Abstract

Patients with pulmonary arterial hypertension (PAH) have a high burden of arrhythmias, including arrhythmias arising from sinus node dysfunction, and the aim of this study was to investigate the effects of PAH on the sinus node. In the rat, PAH was induced by an injection of monocrotaline. Three weeks after injection, there was a decrease of the intrinsic heart rate (heart rate in the absence of autonomic tone) as well as the normal heart rate, evidence of sinus node dysfunction. In the sinus node of PAH rats, there was a significant downregulation of many ion channels and Ca^2+^-handling genes that could explain the dysfunction: HCN1 and HCN4 (responsible for pacemaker current, *I*_f_), Cav1.2, Cav1.3 and Cav3.1 (responsible for L- and T-type Ca^2+^ currents, *I*_Ca,L_ and *I*_Ca,T_), NCX1 (responsible for Na^+^–Ca^2+^ exchanger) and SERCA2 and RYR2 (Ca^2+^-handling molecules). In the sinus node of PAH rats, there was also a significant upregulation of many fibrosis genes that could also help explain the dysfunction: vimentin, collagen type 1, elastin, fibronectin and transforming growth factor β1. In summary, in PAH, there is a remodelling of ion channel, Ca^2+^-handling and fibrosis genes in the sinus node that is likely to be responsible for the sinus node dysfunction.

This article is part of the theme issue ‘The heartbeat: its molecular basis and physiological mechanisms’.

## 1. Introduction

Pulmonary hypertension is diagnosed when the mean pulmonary arterial pressure is raised to greater than 25 mm Hg in human. The increased pulmonary pressure can be the result of various disease processes [1]. In pulmonary arterial hypertension (PAH), there is pathology within the wall of the pulmonary arteries. This may be idiopathic or inherited but is more commonly associated with other underlying conditions such as connective tissue disease (e.g. scleroderma, lupus) [1]. The increase of pulmonary arterial pressure results in an elevation of the right ventricular afterload. The right ventricle is a thin-walled, flow-generating (rather than pressure-generating) structure that is especially sensitive to increases in afterload and PAH ultimately leads to right ventricular hypertrophy, dilatation and failure. Arrhythmias of all kinds are excessively common in patients with PAH [2–7]. Although the most common cause of death in PAH is progressive right heart failure, a significant proportion of patients die unexpectedly from bradycardia, asystole, electromechanical dissociation and ventricular arrhythmias [6]. Arrhythmias in patients with PAH include arrhythmias arising from dysfunction of the cardiac conduction system (sinus bradycardia, sinus tachycardia, first-degree heart block and right bundle branch block) as well as atrial and ventricular arrhythmias [2,4–7]. Rajdev *et al.* [3, p. 185] reviewed arrhythmias in PAH and concluded ‘Ventricular tachycardia is less common, and relative bradycardia is an ominous sign, with bradyarrhythmias frequently observed in the setting of cardiopulmonary arrest’. This suggests that PAH results in cardiac conduction system disease. This is not surprising, because heart failure in general is known to cause dysfunction of the sinus node and the wider cardiac conduction system [8–10] and a significant proportion of end-stage heart failure patients die as a result of bradyarrhythmias [11–16]. The aim of the present study was to investigate sinus node dysfunction in a rat model of monocrotaline (MCT)-induced PAH in which cardiac conduction system dysfunction is recognized as a cause of death [17,18].

## 2. Methods

Sprague-Dawley and Wistar rats were injected with a single dose of 60 mg kg^−1^ MCT to induce PAH. Control rats received a saline injection. Blood pressure was recorded using the tail-cuff method and the electrocardiogram (ECG) and echocardiogram were recorded in the anaesthetized rat. After sacrifice, heart and tissue weights were recorded and serum levels of brain natriuretic peptide (BNP) measured. ECG recordings were made from the isolated Langendorff-perfused heart, and intracellular action potentials were recorded from the isolated sinus node using sharp microelectrodes. Histology was carried out to assess the thickness of the wall of the proximal and distal pulmonary artery, the thickness of the right ventricular free wall, the diameter of right ventricular myocytes, mononuclear cell infiltration in right ventricular tissue, and atelectasis in lung tissue. Quantitative polymerase chain reaction was used to measure messenger RNA (mRNA) expression in the sinus node, right atrium and endocardium of the right ventricular free wall. Data are presented as means ± s.e.m. and differences were assessed by one-way ANOVA, two-way ANOVA or Student's or Welch's *t*-test and were considered significant if *p* < 0.05. *n* refers to number of animals unless stated otherwise. Further details are available in the electronic supplementary material.

## 3. Results

### (a) Development of pulmonary arterial hypertension, hypertrophy of the right side of the heart and sinus node dysfunction

**Figure 1.**
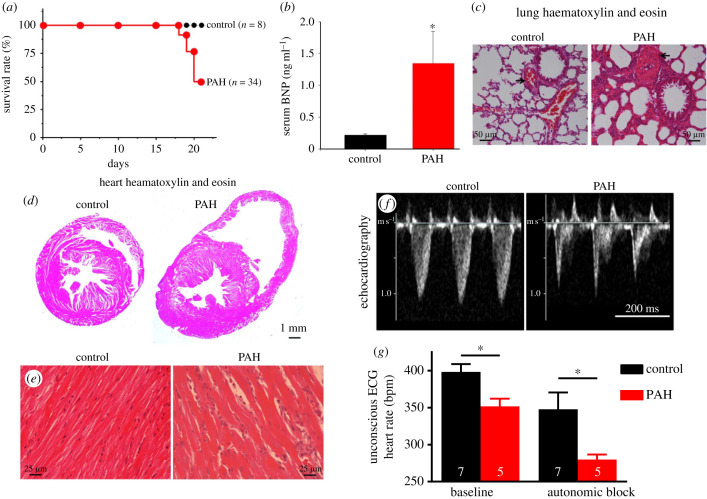
Characterization of the rat model of PAH. (*a*) Survival curves for control (black symbols; *n* = 8) and PAH (red symbols; *n* = 34) rats. Percentage of rats surviving is plotted against time. (*b*) Plasma level of BNP in control (*n* = 5) and PAH (*n* = 4) rats. (*c*) Tissue sections through the middle lobe of the right lung stained with haematoxylin and eosin from control (left) and PAH (right) rats. Arrows highlight pulmonary arteries. (*d*) Short axis sections through control (left) and PAH (right) rat hearts at 21 days after MCT injection. Sections were cut midway between apex and base of the ventricles and stained with haematoxylin and eosin. (*e*) High magnification images of haematoxylin and eosin-stained sections of the right ventricle from control (left) and PAH (right) rats. (*f*) *In vivo* echocardiography images from control (left) and PAH (right) rats showing Doppler traces through the right ventricular outflow tract demonstrating the development of PAH. (*g*) heart rates of control (*n* = 7) and PAH (*n* = 5) rats 23–28 days after MCT injection measured *in vivo* under isoflurane anaesthesia under baseline conditions and after complete autonomic blockade with propranolol (2 mg kg^−1^) and atropine (1 mg kg^−1^). Data are presented as means ± s.e.m.; *Significantly different (*p* < 0.05) from control rats based on Student's unpaired or Welch's *t*-test. See also the electronic supplementary material, table S1.

Twenty-one days after injection of MCT, whereas all control rats were alive, 50% of the MCT-treated rats had died (figure 1*a*), consistent with a report from others [19]. Serum BNP level, an indicator of heart failure, was significantly elevated in the PAH rats (figure 1*b*). After sacrifice, haematoxylin and eosin-stained sections of lung showed that the wall of the pulmonary arteries was significantly thicker in PAH rats than in control rats (figure 1*c*; table 1). In the lung sections from PAH rats, atelectasis was observed and scored significantly higher at 3.3 ± 0.2, whereas in control rats it was not observed and scored at 1 (figure 1*c*; table 1). In the PAH rats (as compared to control rats), the lung weight was significantly increased, but the body weight was significantly reduced; the lung-to-body weight ratio was significantly increased (table 1). Twenty-one days after injection of MCT, systolic and diastolic blood pressures were significantly reduced in the PAH rats (table 1).

**Table 1.** *In vivo* blood pressure, echocardiography data, body and tissue weights and ratios and pathological observations 21 days after MCT injection. (LA, left atrium; LV, left ventricle; PAAT, pulmonary artery acceleration time; PAD, pulmonary artery deceleration; PV_max_, maximum pulmonary artery velocity; RA, right atrium; RV, right atrium.)

				control (*n* = 4–8)	PAH (*n* = 6–12)
blood pressure (mm Hg)	systolic blood pressure			115 ± 4	105 ± 10^c^
	diastolic blood pressure			91 ± 3	84 ± 6^c^
echocardiography	LV septal wall, diastole (cm)			0.15 ± 0.018	0.19 ± 0.006
	LV internal diameter, diastole (cm)			0.75 ± 0.035	0.64 ± 0.070
	LV posterior wall, diastole (cm)			0.16 ± 0.009	0.16 ± 0.016
	LV septal wall, systole (cm)			0.33 ± 0.014	0.29 ± 0.020
	LV internal diameter, systole (cm)			0.31 ± 0.043	0.30 ± 0.058
	LV posterior wall, systole (cm)			0.30 ± 0.020	0.26 ± 0.020
	PAAT (ms)			31.00 ± 4.50	19.17 ± 0.97^*p* = 0.06^
	PAD (m s^−1^)			9.97 ± 0.72	29.12 ± 6.04^a^
	PV_max_ (m s^−1^)			0.96 ± 0.057	0.87 ± 0.063
	RV wall, diastole (cm)			0.06 ± 0.003	0.11 ± 0.015^b^
	RV internal dimension, diastole (cm)			0.29 ± 0.022	0.29 ± 0.017
	RV wall, systole (cm)			0.12 ± 0.012	0.14 ± 0.025
	RV internal dimension, systole (cm)			0.13 ± 0.016	0.19 ± 0.062
weight (g)	body			365.1 ± 12.0	279.6 ± 7.4^c^
	heart			1.01 ± 0.028	1.052 ± 0.026
	RA			0.043 ± 0.002	0.058 ± 0.003^c^
	LA			0.030 ± 0.002	0.023 ± 0.001^c^
	RV			0.172 ± 0.005	0.340 ± 0.010^c^
	LV			0.765 ± 0.023	0.630 ± 0.022^c^
	lungs			1.19 ± 0.05	2.32 ± 0.11^c^
weight ratio (%)	tissue/heart	RA/heart		4.3 ± 0.2	5.5 ± 0.3^c^
		LA/heart		2.9 ± 0.1	2.2 ± 0.1^c^
		RV/heart		17.1 ± 0.5	32.5 ± 0.8^c^
		LV/heart		75.7 ± 0.4	59.8 ± 1.0^c^
	tissue/body	heart/body		0.3 ± 0.0	0.4 ± 0.0^c^
		RA/body		0.012 ± 0.001	0.018 ± 0.001^c^
		LA/body		0.008 ± 0.000	0.009 ± 0.000
		RV/body		0.047 ± 0.002	0.138 ± 0.004^c^
		LV/body		0.210 ± 0.005	0.238 ± 0.006^c^
		lung/body		0.4 ± 0.0	0.9 ± 0.1^c^
pathological observations	middle lobe of the right lung	pulmonary artery wall thickness (μm)	proximal	46.5 ± 5.0	61.4 ± 3.3^c^
			distal	19.5 ± 2.8	32.1 ± 3.3^c^
		atelectasis^d^		1 ± 0.0	3.3 ± 0.2^c^
	RV	thickness of free wall (mm)		0.9 ± 0.0	1.1 ± 0.0^c^
		myocyte diameter (μm)		8.5 ± 0.6	17.9 ± 1.1^c^
		mononuclear cell infiltration^d^		1 ± 0.0	2.1 ± 0.3^c^

^a^Significantly different from control group (*p* < 0.05; Student's *t*-test).

^b^Significantly different from control group (*p* < 0.005; Student's *t*-test).

^c^Significantly different from control group (*p* < 0.05; two-way ANOVA).

^d^Scoring: 1, negative; 2, weakly positive; 3, positive; 4, strongly positive.

Although the heart weight was not different between PAH and control rats, the heart-to-body weight ratio was significantly increased in PAH rats (table 1). Interestingly, the four cardiac chambers were differentially affected: in PAH rats, the right atrium and right ventricle weights were significantly increased (as were the right atrium-to-body weight and right ventricle-to-body weight ratios), but the left atrium and left ventricle weights were significantly decreased (although left ventricle-to-body weight ratio was significantly increased; table 1). Consistent with the change in right ventricular weight, the thickness of the right ventricle free wall assessed from haematoxylin and eosin-stained short axis sections through the ventricles (figure 1*d*) was significantly increased in PAH rats (table 1). The haematoxylin and eosin-stained sections also showed an increase in the diameter of the right ventricle (figure 1*d*). The right ventricle sections showed that the myocytes were significantly larger in diameter and more poorly organized in PAH rats (figure 1*e*; table 1). Finally, the right ventricle sections from PAH rats (but not control rats) showed infiltration of mononuclear cells (stained purple with haematoxylin) between myocytes (figure 1*e*). Infiltration of mononuclear cells was scored and was significantly higher in PAH rats (table 1).

In surviving animals 21 days after injection of MCT, echocardiography showed the profile of pulmonary artery blood flow to be more spike-like (figure 1*f*). The pulmonary artery acceleration time tended to be shorter (there was a 38% reduction in the pulmonary artery acceleration time; *p* = 0.06) and the initial phase of pulmonary artery deceleration was faster (there was a 190% increase in the pulmonary artery deceleration; *p* = 0.01; table 1). These changes are validated measures of pulmonary hypertension [20]. There was also evidence of hypertrophy of the right ventricle with a significant 23% increase in diastolic right ventricle wall thickness (table 1). No other echocardiography parameters showed significant differences; in particular, there was no evidence of hypertrophy of the left ventricle (table 1). This pattern of hypertrophy is expected: PAH will place stress on the right side of the heart and, therefore, right-sided hypertrophy is expected. On the other hand, if the right ventricular output is reduced, the loading of the left side of the heart will be reduced and, therefore, left-sided hypertrophy is not expected. This is the pattern observed (table 1) and this same pattern has been reported in PAH patients [21].

**Figure 2.**
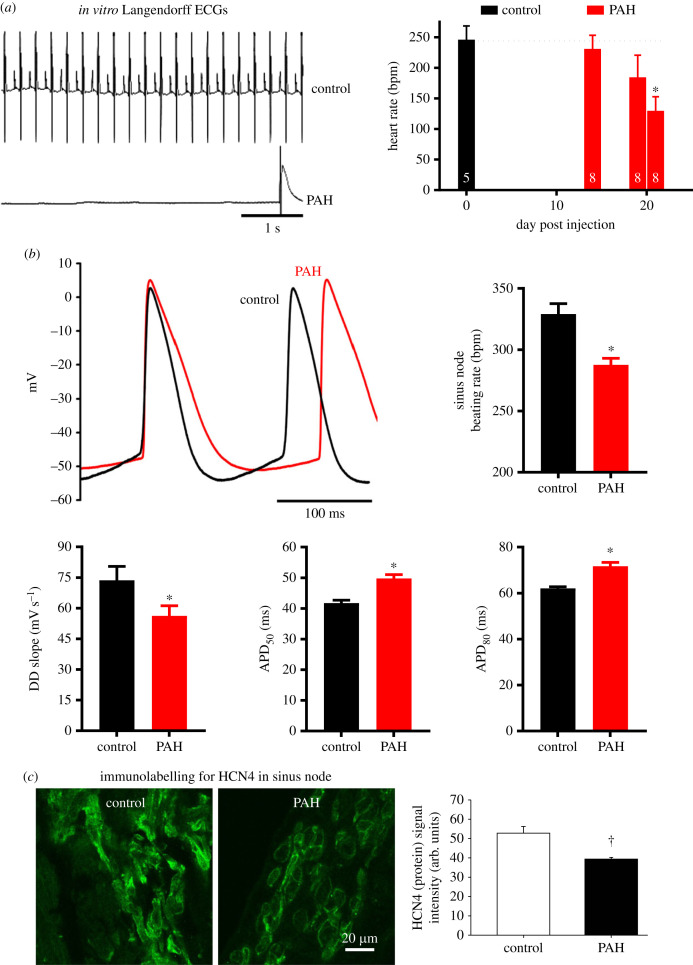
Intrinsic bradycardia and action potential remodelling in PAH. (*a*, left) ECG-like electrogram recordings from Langendorff-perfused hearts from control and PAH rats showing an extreme case of bradycardia (6.1 beats min^−1^) in PAH on day 21 following MCT injection. (*a*, right) Bar chart showing time course of changes in heart rate (measured in the Langendorff-perfused heart) following MCT injection (right). Dotted line indicates the control level. Asterisk, significantly different (*p* < 0.05; one-way ANOVA followed by Dunnett's multiple comparisons test; *n* numbers shown within bars) from data from control rats. (*b*) Intracellular action potentials from the sinus node of control and PAH rats 23–28 days after MCT injection (top left) and bar charts showing sinus node beating rate, slope of DD and APD at 50% and 80% repolarization in the sinus node of control (*n* = 17 impalements at different sites in six hearts) and PAH (*n* = 26 impalements at different sites in nine hearts) rats (top right and bottom). Asterisk, significantly different (*p* < 0.05; Student's unpaired or Welch's *t*-test). See also the electronic supplementary material, table S1. (*c*) Expression of HCN4 protein in sinus node in control and PAH rats. The left-hand panels show sinus node sections from control and PAH rats immunolabelled for HCN4 (green signal) and the right-hand panel shows the abundance of HCN4 protein in the sinus node (control, *n* = 4 rats; PAH, *n* = 3 rats; 15–17 images from each rat). Dagger symbol, significantly different (*p* < 0.05) from control based on Student's *t*-test.

Following 23–28 days after injection of MCT, the ECG was recorded *in vivo* from the anaesthetized rat, and this showed a significant decrease in the heart rate in PAH (figure 1*g*). *In vivo* the heart rate is influenced by autonomic tone; the *intrinsic heart rate* (in the absence of autonomic tone) was measured in three ways. First, in the same cohort of rats, it was measured *in vivo* during complete autonomic blockade following injection of propranolol and atropine; the intrinsic heart rate measured in this way was significantly lower in the PAH rats (figure 1*g*). Second, 21 days after the injection of MCT, it was measured *in vitro* in the denervated Langendorff-perfused heart (figure 2*a*). Figure 2*a* shows ECG-like electrograms recorded from hearts from control and PAH rats; whereas the control heart beat normally, the PAH heart was largely quiescent. The bar chart on the right of figure 2*a* shows the mean intrinsic heart rate in the 21 days following the MCT injection. There was a significant decrease in the intrinsic heart rate in the final days (figure 2*a*). Finally, the intrinsic heart rate was measured in denervated isolated sinus node preparations; in these experiments, the intracellular action potential was recorded using sharp microelectrodes (figure 2*b*). The typical action potentials from control and PAH rats in figure 2*b* show that in PAH there was a decrease in the sinus node beating rate (equivalent to the intrinsic heart rate) because of a decrease in the slope of diastolic depolarization (DD), and there was also an increase in action potential duration (APD); this is confirmed by the mean data shown in figure 2*b*. The three types of the experiment demonstrate that there is a decrease in the intrinsic heart rate in PAH and this is evidence of sinus node dysfunction in PAH. To determine the mechanism underlying the sinus node dysfunction, the expression of ion channel and fibrosis genes in the sinus node was determined.

### (b) Downregulation of funny channels during pulmonary arterial hypertension

**Figure 3.**
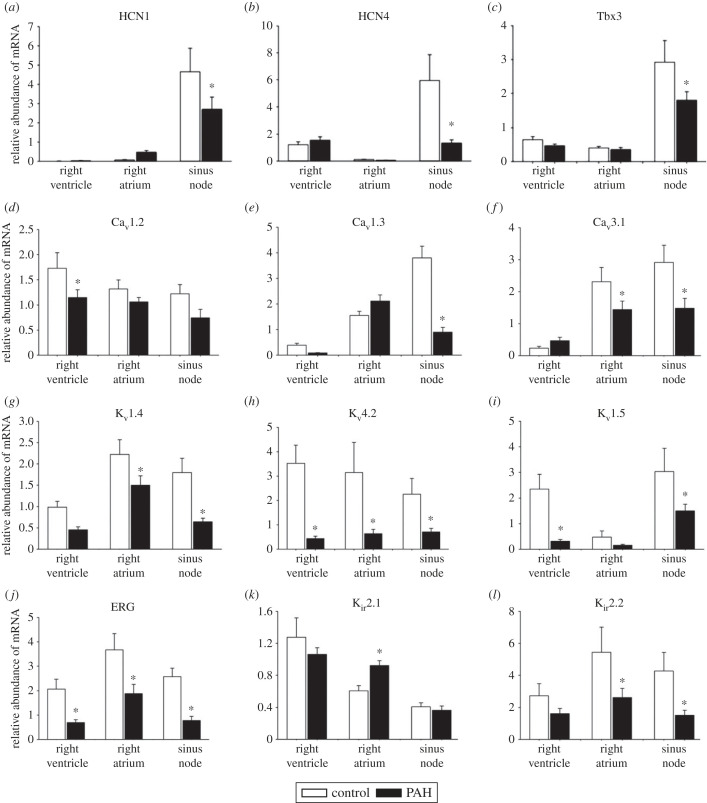
Remodelling of ion channels and Tbx3 in the sinus node in PAH. (*a*–*l*) Expression of HCN1 and HCN4 mRNAs (*a*,*b*), Tbx3 mRNA (*c*) and various Ca^2+^ and K^+^ channel mRNAs (*d*–*l*). In this and similar figures, panels show the relative abundance of mRNA calculated using the ΔΔCT technique or absolute abundance of mRNA calculated using the ΔCT technique in the right ventricle, right atrium and sinus node of control (*n* = 8) and PAH (*n* = 12) rats. Asterisk, significantly different (*p* < 0.05) from corresponding data from control rats based on two-way ANOVA.

Cryosections through the sinus node were cut from control and PAH rat hearts. Some sections were histologically stained to identify the sinus node [22,23] and others immunolabelled for HCN4 protein (figure 2*c*). Velocity software (Improvision, UK) was used to measure the signal intensity (in arbitrary units) for HCN4 protein, which was expressed in the cell membrane. The HCN4 protein signal was significantly decreased in the sinus node during PAH (figure 2*c*). HCN1 and HCN4 channels are primarily responsible for *I*_f_, a major pacemaker current in the sinus node. As expected, HCN1 and HCN4 mRNA expression was high in the sinus node and low in the working myocardium (figure 3*a*). HCN1 and HCN4 mRNA expression was significantly decreased in the sinus node (but not in the working myocardium) during PAH (figure 3*a*,*b*). Tbx3 is a transcription factor that plays a key role in the regulation of the expression of ion channels, including HCN1 and HCN4, in the sinus node [24]. The expression profile of Tbx3 mRNA was the same as that of HCN1 and HCN4 mRNAs: it was highly expressed in the sinus node, but not in the working myocardium, and during PAH it was significantly decreased in the sinus node (figure 3*c*).

### (c) Dysregulation of ion channels during pulmonary arterial hypertension

The Na^+^ channel, Na_v_1.5, is responsible for the Na^+^ current, *I*_Na_, and the Ca^2+^ channels, Ca_v_1.2, Ca_v_1.3 and Ca_v_3.1, are responsible for the L- and T-type Ca^2+^ currents, *I*_Ca,L_ and *I*_Ca,T_, respectively. Whereas *I*_Na_ plays a limited role in the sinus node, *I*_Ca,L_ and *I*_Ca,T_ play a central role in sinus node pacemaking and the upstroke and plateau of the sinus node action potential. There were no significant changes in Na_v_1.5 in PAH (electronic supplementary material, table S2). In control rats, the abundance of Ca_v_1.3 and Ca_v_3.1 mRNAs was right ventricle < right atrium < sinus node, whereas Ca_v_1.2 mRNA tended to be distributed in the opposite manner (figure 3*d*–*f*). During PAH, there was a significant downregulation of Ca_v_1.2 mRNA in the right ventricle, a significant downregulation of Ca_v_1.3 mRNA in the sinus node and a significant downregulation of Ca_v_3.1 mRNA in the right atrium and sinus node (figure 3*d*–*f*).

Reduced transient outward K^+^ current (*I*_to_) is the most consistent ionic current change in failing hearts [25]. In the rat, K_v_4.2 is the major K^+^ channel responsible for *I*_to_ [26], but K_v_1.4 and K_v_4.3 may also play a role [27]. K_v_1.5, Ether-à-go-go-related gene (ERG) and K_v_LQT1 are responsible for the ultra-rapid, rapid and slow delayed rectifier K^+^ currents, *I*_K,ur_, *I*_K,r_ and *I*_K,s_, respectively. All of these K^+^ currents are involved in action potential repolarization. During PAH, there was a significant downregulation of K_v_1.4, K_v_4.2, K_v_1.5 and ERG mRNAs in most tissues (figure 3*g*–*j*); K_v_4.3 and K_v_LQT1 mRNAs were unchanged (data not shown). K_ir_2 channels are responsible for the background inward rectifier K^+^ current, *I*_K,1_, which is responsible for the stable resting potential in the working myocardium. The density of *I*_K,1_ is ventricle > atrium > sinus node—the low density of *I*_K,1_ in the sinus node allows pacemaking to occur. As expected K_ir_2.1 mRNA was distributed in the same manner, although K_ir_2.2 was not (figure 3*k*,*l*). During PAH, there was significant upregulation of K_ir_2.1 mRNA in the right atrium, but a significant downregulation of K_ir_2.2 mRNA in the right atrium and sinus node (figure 3*k*,*l*). K_ir_3.4 is one of the channel subunits responsible for the ACh-activated K^+^ current, *I*_K,ACh_, and it was unchanged in PAH (data not shown). K_ir_6 channels together with the sulfonylurea receptor (SUR) β-subunits are responsible for the ATP-sensitive current, *I*_K,ATP_. *I*_K,ATP_ serves a cardioprotective role in ischaemia [28]. Reconstitution experiments suggested that K_ir_6.2 and SUR2A are responsible for *I*_K,ATP_ [29], although it is now thought that K_ir_6.1 and SUR1 also play a role [27,30,31]. In PAH, K_ir_6.2 mRNA was significantly downregulated in all tissues, whereas K_ir_6.1 mRNA was not (electronic supplementary material, figure S2). In PAH, SUR1 mRNA was significantly downregulated in all tissues, whereas SUR2 mRNA was only significantly downregulated in the right atrium and sinus node (electronic supplementary material, figure S2).

### (d) Changes of connexins and Ca^2+^-handling molecules during pulmonary arterial hypertension

**Figure 4.**
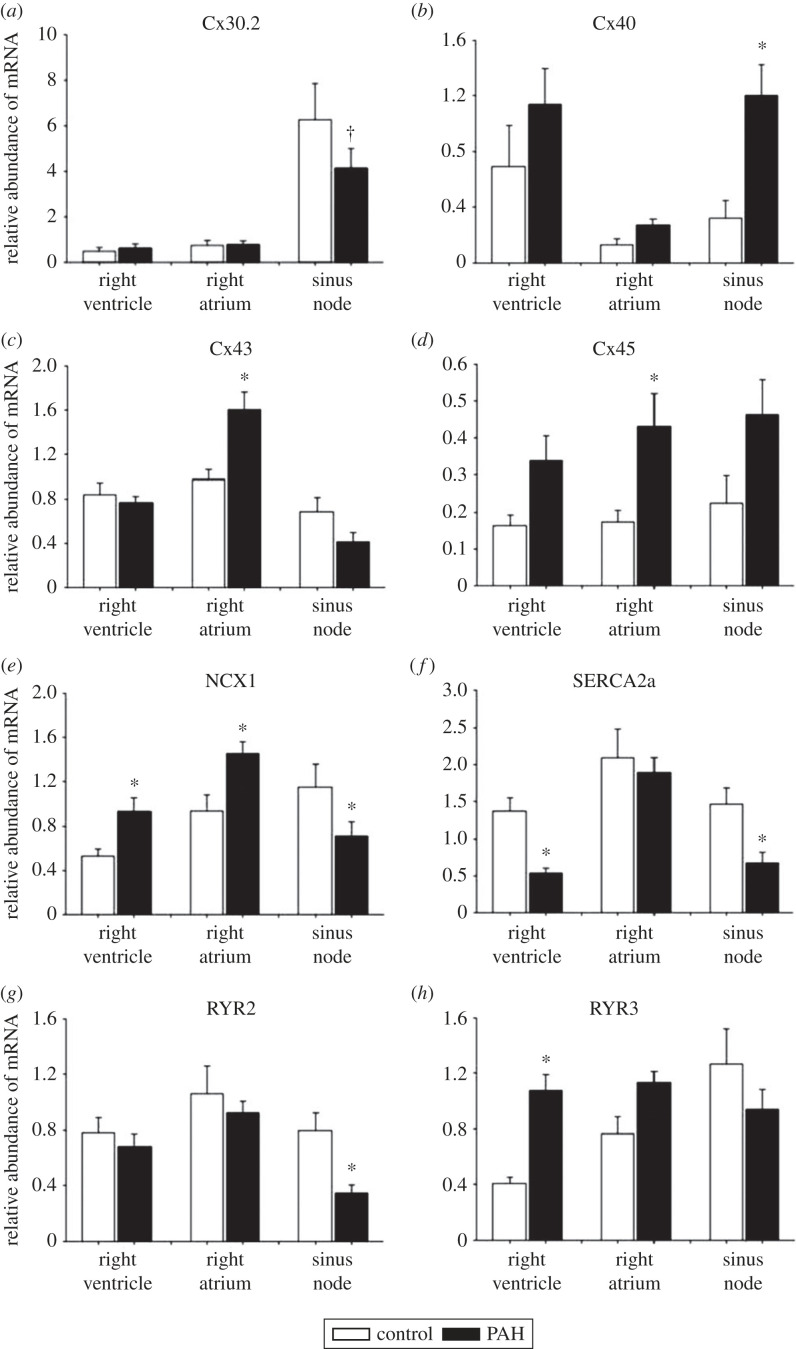
Remodelling of connexins (*a*–*d*) and Ca^2+^-handling molecules (*e*–*h*) in the sinus node in PAH. Asterisk and dagger symbols: significantly different (*p* < 0.05) from corresponding data from control rats based on two-way ANOVA (asterisk) or Student's *t*-test (dagger).

Gap junctions, comprised connexins, are responsible for electrical coupling between cardiac myocytes. Four principal connexins are expressed in cardiac myocytes: Cx30.2, Cx40, Cx43 and Cx45. There was a significant upregulation of connexin mRNAs in some tissues, but a significant downregulation of Cx30.2 in the sinus node (figure 4*a*–*d*).

Spontaneous diastolic release of intracellular Ca^2+^ from the sarcoplasmic reticulum (SR) via the ryanodine receptor and activation of the electrogenic Na^+^–Ca^2+^ exchanger has been implicated in the pacemaker activity of the sinus node [32]. It is referred to as the ‘Ca^2+^ clock’ mechanism of pacemaking. Four Ca^2+^-handling molecules were investigated: NCX1 (responsible for the Na^+^–Ca^2+^ exchanger), SERCA2 (SR Ca^2+^ uptake pump), RYR2 (main ryanodine receptor) and RYR3 (alternative ryanodine receptor). During PAH, in the sinus node (but not necessarily in other tissues), there was a significant downregulation of NCX1, SERCA2 and RYR2 mRNAs (figure 4*e*–*h*). In the right ventricle, there was a significant upregulation of NCX1 mRNA and significant downregulation of SERCA2 mRNA (figure 4*e*,*f*) as has been observed in other heart failure models [33].

### (e) Upregulation of fibrosis genes during pulmonary arterial hypertension

**Figure 5.**
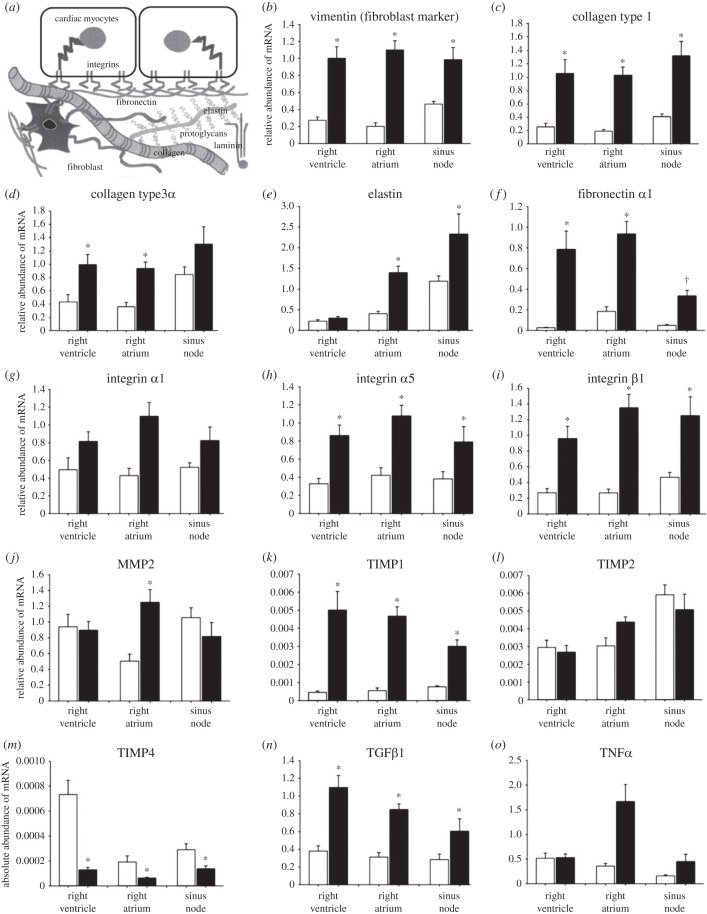
Upregulation of fibrosis genes in the sinus node in PAH. (*a*) Schematic diagram of cardiac tissue including myocytes and interstitium; modified from MacKenna *et al.* [34]. (*b*–*o*) Expression of various interstitial component mRNAs in control (open bars) and PAH (closed bars) (*b*–*d*).

To test if there is a remodelling of the interstitium of the sinus node during PAH, the abundance of mRNAs for important interstitial components was measured. The two main components of the interstitium are fibroblasts and the extracellular matrix (principally collagens; figure 5*a*) [35]. Vimentin is expressed by fibroblasts (as well as other cell types) and vimentin mRNA was significantly upregulated during PAH (figure 5*b*) [36]. These data suggest that there was a PAH-dependent increase in the number of fibroblasts in the sinus node and elsewhere. Collagens are structural proteins made by the fibroblasts (figure 5*a*). Types 1 and 3 collagens constitute 85% and 11%, respectively, of the total collagen present in the interstitium in the heart [37]. Both collagen types were significantly upregulated in most tissues during PAH (figure 5*c*,*d*). Another structural protein of the extracellular matrix is elastin (figure 5*a*) and elastin mRNA was more abundant in the sinus node than in the working myocardium (figure 5*e*). As in the case of the collagen mRNAs, there was a PAH-dependent upregulation in elastin mRNA in the sinus node and right atrium at least (figure 5*e*). Fibronectin α1 is an adhesive protein in the extracellular matrix (figure 5*a*). During PAH, there was a significant upregulation of fibronectin α1 mRNA in all tissues (figure 5*f*). Integrins are cell membrane receptors, which bind to the extracellular matrix (figure 5*a*). Three integrins were measured (α1, α5 and β1), all of which are important in the heart [37]. During PAH, there was a significant upregulation of the integrin subunits at the mRNA level in most tissues (figure 5*g*–*i*). Metalloproteinases (MMPs) are a group of enzymes that catalyse the degradation of the extracellular matrix including collagens [37]. They are, therefore, anti-fibrotic. MMP2 is an important MMP in rat heart [37]. The abundance of MMP2 mRNA was similar in all tissues and there was a significant PAH-dependent upregulation in MMP2 mRNA in the right atrium (figure 5*j*) as has been observed by others in the working myocardium in various species and heart failure models [37]. MMP activity is inhibited by endogenous inhibitors, the tissue inhibitors of metalloproteinases (TIMPs) [37]. The TIMPs are, therefore, profibrotic. Four TIMPs (TIMP1–TIMP4) are known to exist in vertebrates and all four are found within the myocardium [37]; we studied TIMP1, TIMP2 and TIMP4 (figure 5*k*–*m*). Within the heart, TIMP2 is constitutively expressed in most cell types, whereas TIMP1 is inducible in response to signals such as proinflammatory cytokines [37]. TIMP4 is reported to be the most abundant TIMP within the heart and is cardiac-specific [37]. However, in the sinus node, at the mRNA level, TIMP2 ≫ TIMP1 ≈ TIMP4 in the control rats and TIMP1 ≈ TIMP2 ≫ TIMP4 mRNA in the PAH rats (figure 5*k*–*m*). There is a relatively low degree of specificity for substrates between the TIMPs. For example, MMP2 can be inhibited by TIMP1–TIMP4. During PAH, in all tissues, there was a significant upregulation of TIMP1 and downregulation of TIMP4; TIMP2 showed no changes (figure 5*k*–*m*). An upregulation of TIMP1 and downregulation of TIMP4 has been observed by others in the working myocardium in various species and heart failure models [37]. Transforming growth factor β1 (TGFβ1) and tumour necrosis factor α (TNFα) are fibrotic agents: they promote the formation of extracellular matrix including collagens (regulated at the transcriptional level) [38]. The abundance of TGFβ1 and TNFα mRNAs was similar in all tissues in control animals (figure 5*n*,*o*). There was a significant, PAH-dependent upregulation of TGFβ1 mRNAs in all tissues, whereas there was only a significant upregulation of TNFα mRNA in the right atrium (figure 5*n*,*o*). In summary, there was a PAH-dependent upregulation of fibrosis genes (figure 5).

## 4. Discussion

For the first time, to our knowledge, this study has shown that PAH is associated with dysfunction of the sinus node because of an adverse remodelling of ion channels and an upregulation of fibrosis genes.

### (a) Heart failure and bradyarrhythmias

A significant proportion of patients with PAH die as a result of an arrhythmia [6]. Arrhythmias in PAH patients include those arising from dysfunction of the cardiac conduction system [2,7]. PAH also results in heart failure. Up to 80% of heart failure patients in general die of sudden cardiac death [39], i.e. possibly of an arrhythmia; 41–68% of the sudden cardiac deaths of end-stage heart failure patients are reported to be the result of bradyarrhythmias [11,12,16]. The bradyarrhythmias could be the result of dysfunction of the cardiac conduction system: in patients, there is good evidence that heart failure is associated with dysfunction of the sinus node [12], atrioventricular node [40,41] and the His–Purkinje system [42]. Animal studies (using dog, rabbit and rat) have confirmed that heart failure (induced by rapid ventricular pacing, volume and pressure overload or myocardial infarction) is associated with dysfunction of the cardiac conduction system (slowing of the intrinsic pacemaker activity of sinus node; slowing of conduction through the atrioventricular node and His–Purkinje system) [9,10,27,43–48].

### (b) Sinus node dysfunction in pulmonary arterial hypertension and underlying mechanisms

In the well-established rat model of PAH, we observed a decrease in the intrinsic pacemaker activity of the sinus node, evidence of sinus node dysfunction (figure 2). The time course of the decrease in the intrinsic pacemaker activity (figure 2*a*) was similar to that of mortality (figure 1*a*); this suggests that it occurs late in the disease progression. This is consistent with observations on heart failure patients: bradyarrhythmic deaths are more frequent with more severe heart failure (New York Heart Association, class IV) [11]. During PAH, in the sinus node, we observed a downregulation of many ion channels at the mRNA level and the ion channels affected are summarized in the electronic supplementary material, table S2. If the changes are duplicated at the protein level (as we have shown for HCN4; figure 2*c*), the decreases in HCN1, HCN4, Ca_v_1.3, Ca_v_3.1, K_v_1.4, K_v_4.2, K_v_1.5, ERG, K_ir_2.2 and K_ir_6.2 are expected to result in decreases of *I*_f_, *I*_Ca,L_, *I*_Ca,T_, *I*_to_, *I*_K,ur_, *I*_K,r_, *I*_K,1_ and *I*_K,ATP_. It is well established that decreases in *I*_f_, *I*_Ca,L_, *I*_Ca,T_ and *I*_K,r_ slow pacemaking. A decrease in K^+^ currents (*I*_to_, *I*_K,ur_, *I*_K,r_, *I*_K,1_ and *I*_K,ATP_) can explain the prolongation of the action potential shown in figure 2*b*. A decrease in *I*_K,1_ could result in a depolarization during diastole such as that shown in figure 2*b*. The Ca^2+^-clock plays an important role in pacemaking and three key Ca^2+^ clock molecules (NCX1, SERCA2a and RYR2) were downregulated at the mRNA level and if the changes are duplicated at the protein level they are expected to further slow pacemaking. There was a change in gap junction channels, Cx30.2 and Cx40, at the mRNA level, but both connexins are poorly expressed in the sinus node and the significance of the change is not known. In the sinus node in PAH, we also observed widespread upregulation of fibrosis transcripts: in vimentin mRNA (fibroblast marker), in mRNA for components of the extracellular matrix (collagen 1, elastin, fibronectin), in mRNA for receptors that mediate attachment between myocytes and the extracellular matrix (integrin α5, integrin β1), in TIMP1 mRNA, and in mRNA for the fibrotic agent, TGFβ1 (electronic supplementary material, table S2). A decrease in TIMP4 mRNA was observed, but the increase in TIMP1 mRNA was greater than the decrease in TIMP4 mRNA (figure 5*k*,*m*).

What is the driving force behind the remodelling of the sinus node? Tbx3 is a transcription factor that is expressed in the nodal tissues of the heart (but not in the working myocardium; figure 3*c*) and is important for the embryonic development of the nodal tissues [49]. Hoogaars *et al.* [49] showed that ectopic expression of Tbx3 in the atrial muscle of the embryonic mouse heart converts the atrial muscle to a sinus node phenotype. Hoogaars [50] has shown that Tbx3 controls the expression of a wide range of ion channels, including HCN1 and HCN4 [24]. Based on the work of Hoogaars [24,50], a fall of Tbx3 (as observed in the sinus node during PAH—figure 3*c*) would be expected to result in a fall in HCN1 and HCN4 and figure 3*a*,*b* shows that such a fall was observed and these are clues concerning the driving force behind the remodelling of the sinus node. However, other transcription factors (and micro-RNAs) are also likely to be involved. What is the precipitating step? Stretch of the right atrium is one possibility—table 1 shows that the weight of the right atrium was significantly increased in PAH rats.

### (c) Possible cause of death in pulmonary arterial hypertension

Is sinus node dysfunction the cause of death in PAH? It is possible, because bradyarrhythmias are an important cause of death in heart failure (see above). Here, using a rat MCT model, we show marked sinus node remodelling and dysfunction in PAH. There is also evidence of atrioventricular node dysfunction in PAH patients [5], and our work [51] and the work of others [17,18] show that there is atrioventricular node dysfunction in the rat MCT model. Watkinson *et al*. [17] and Chi *et al*. [18] estimated that approximately 38% of MCT-injected rats die as a result of severe atrioventricular node dysfunction and heart block. Thus, cardiac conduction system remodelling may be an important cause of death in PAH.

## Data Availability

The data are provided in the electronic supplementary material [52].
